# Postoperative Serum Levels of sCD26 for Surveillance in Colorectal Cancer Patients

**DOI:** 10.1371/journal.pone.0107470

**Published:** 2014-09-11

**Authors:** Loretta De Chiara, Ana M. Rodríguez-Piñeiro, Oscar J. Cordero, Lidia Vázquez-Tuñas, Daniel Ayude, Francisco J. Rodríguez-Berrocal, María Páez de la Cadena

**Affiliations:** 1 Departamento de Bioquímica, Genética e Inmunología, Universidad de Vigo, Vigo, Pontevedra, Spain; 2 Departmento de Bioquímica y Biología Molecular, Edificio CIBUS, Campus Vida, Universidad de Santiago de Compostela, Santiago de Compostela, A Coruña, Spain; 3 Servicio de Oncología, Complejo Hospitalario Universitario de Vigo, Vigo, Pontevedra, Spain; Sapporo Medical University, Japan

## Abstract

One of the main aims of the follow-up after curative resection of colorectal cancer is the early detection and treatment of tumor recurrence. We previously demonstrated decreased preoperative soluble CD26 (sCD26) levels in serum from colorectal cancer patients. We extended now the study to investigate if sCD26 levels in postoperative serum serve as marker of recurrence of the disease during surveillance. Soluble sCD26 was measured in pre- and postoperative serum samples of 43 patients with primary colorectal cancer. Carcinoembryonic antigen, carbohydrate antigen 19.9 and 72.4 levels were also measured during surveillance. The average follow-up period was 41.8±20.8 months. sCD26 levels during follow-up showed well-defined patterns in patients without disease (n = 28), and in patients with tumor persistence (n = 2), local recurrence (n = 3) or distant metastasis (n = 10). Disease-free patients showed stable levels between 460–850 ng/mL during follow-up, while high (over 850 ng/mL) and unstable sCD26 levels were found before recurrence was diagnosed. The mean maximum/minimum sCD26 ratios during surveillance were 1.52, 2.12 and 2.63 for patients with no recurrence, local recurrence and metastasis, respectively (*p* = 0.005). From the cut-off obtained from a receiver operator characteristics (ROC) curve built with the maximum/minimum sCD26 ratios and the upper and lower cut-offs of sCD26, we were able to discriminate patients with and without recurrent disease. We propose that the measurement of serum sCD26 during the follow-up of patients diagnosed of colorectal cancer could be valuable for the early detection of local and distant recurrence. A large, randomized, prospective trial should be performed to confirm our findings.

## Introduction

At the time of diagnosis, about 75% of colorectal cancer (CRC) patients have the tumor confined to a portion of the bowel or to regional lymph nodes, and can be referred for curative resection. Unfortunately, 30–50% of those patients develop recurrence, 90% during the first 5 years after treatment [1], [2].

One of the aims of the follow-up after curative resection in CRC patients is to improve the outcome by early detection and treatment of recurrence. Thus postoperative surveillance must identify asymptomatic recurrences for the early detection of locally persistent tumors or metastases, so that further curative treatment can be initiated and the survival rates improved. Consequently, surveillance strategies require effective means for identifying residual or recurrent disease. In general, meta-analyses and reviews agree that a more intensive follow-up contributes to an overall survival benefit [1]–[8].

Many different methods have been proposed for the follow-up of CRC patients, which can be subdivided into three categories: laboratory tests, as determination of carcinoembryonic antigen (CEA) serum levels, other markers as the carbohydrate antigens (CA), or liver enzymes; image tests, as ultrasound, X-ray or computed tomography; and endoscopies. Compared to other available diagnostic modalities, serial CEA determinations appear to be the most sensitive for the detection of early recurrent disease [6]–[8]. However, the current serum markers used to detect cancer recurrence (CEA, TPS, CA-19.9 and CA-72.4) are not very accurate and, in general, give rise to a considerable number of false negatives and positives [9]–[10]. Therefore additional testing is usually necessary to confirm the recurrence, generating inconveniences for the patients and elevating the healthcare costs, because some of the techniques are expensive and have not been shown to be cost-effective [11].

The protease CD26, or dipeptidyl peptidase IV (DPP-IV), EC 3.4.14.5, is a cell surface-associated glycoprotein, expressed on a variety of cell types including melanocytes, epithelial cells and lymphocytes [12]. Significant levels of its soluble form (sCD26) exist in plasma/serum and other biological fluids [13], [14]. In previous studies we detected that patients with primary CRC had decreased sCD26 levels in *preoperative* serum, and showed its value as diagnostic and prognostic marker for CRC [15] and advanced adenomas [16]. Two independent studies confirmed that sCD26 is among the best candidates for future blood-based tests for early diagnosis, alone or in combination with fecal immunochemical test (FIT) [17], [18].

We had noted in our previous work that the diagnostic value of sCD26 was worse for Dukes’ stage D patients, showing very high levels in some individuals [15]. Here we designed a pilot study to investigate if sCD26 level measured during the follow-up of CRC patients (*postoperative* sera) is useful as marker of recurrence or regression of the disease during cancer surveillance.

## Materials and Methods

### Population

Forty-three patients with primary CRC were studied, including 28 men (65.1%) and 15 women (34.9%), with a mean age of 66.4±10.4 years (median = 66) (Table S1). Forty-one patients (95.3%) were treated by curative resection (complete tumor removal *en bloc* with a portion of normal bowel, mesenteric and regional lymph nodes), and 2 (4.7%) through palliative surgery. According to Dukes’ stage, 16.3% of the tumors were classified as A, 46.5% as B, 25.6% as C, and 11.6% as D. Regarding the degree of differentiation, 83.3% were moderately differentiated and 14.3% were poorly differentiated, while 2.4% were well differentiated. The localization of the primary tumors was: 4 in cecum (9.3%), 1 in ascending colon (2.3%), 7 in hepatic flexure (16.2%), 2 in splenic flexure (4.7%), 2 in descending colon (4.7%), 10 in sigma (23.3%), 5 in the rectum-sigma union (11.6%), 10 in rectum (23.3%), and synchronic tumors in ascending colon and cecum (2.3%), and in transverse colon and cecum (2.3%).

All patients were monitored at Complejo Hospitalario Universitario de Vigo (Spain). The study followed the clinical-ethical practices of the Spanish Government and the Helsinki Declaration, and was approved by the Galician Ethical Committee for Clinical Research. Written Informed consent was obtained and anonymity warranted. The clinical information collected included Dukes’ stage, primary tumor site, type of resection, cancer progression and chemotherapy treatment.

The standard follow-up procedure consisted on a medical examination every 4 months during the first year, and every 6 months after that. According to the oncologist’s criteria, patients received chemotherapy consisting on 5-fluorouracil, and in some cases irinotecan.

### Sample collection and preparation

Preoperative blood samples were collected from near but not all the patients. Postoperative blood samples were collected at several time points, which were not the same for each patient. Blood was allowed to coagulate at room temperature and centrifuged at 2000 *g* for 15 minutes. Sera were stored at −85°C until used.

### Determination of the sCD26 levels

The concentration of sCD26 was analyzed with the Human Soluble CD26 ELISA Kit (eBioscience; Vienna, Austria) in duplicate. Based on our previous results with this kit [15], [16], [19] sCD26 values between 460–850 ng/mL were considered as normal levels. The lower limit was established in accordance with the 460 ng/mL cut-off suggested for our cohort of patients under risk for CRC or with related colorectal pathologies [16], while for the upper limit we hypothesized a 850 ng/mL cut-off for pathological individuals (with higher levels) from our preliminary results with CRC metastatic patients and tumor-resected patients (reviewed in [20]).

### Determination of the CEA, CA-19.9 and CA-72.4 levels

CEA and CA-72.4 were analyzed in serum using the electrochemoluminescent immunoassay Roche Elecsys System, and measured with a Modular Analytics E170 analyzer (Roche Diagnostics). CA-19.9 was determined using the TRACE BRAHMS CA 19-9 KYPTOR immunoassay (Thermo Scientific) and measured in a Kryptor analyzer (CIS bio international). Normal values were <5 ng/mL for CEA, <7 U/mL for CA-72.4, and <40 U/mL for CA-19.9 [7]–[10].

### Data analysis

All the measurements included were posterior to 2 months after surgery to allow for normalization of the marker. Statistical analyses were performed with the SPSS package (v.19.0); tests were two-sided; *p*-values<0.05 were considered significant. Chi-square or Fischer's exact tests were done with contingency tables. The analysis of more than two independent samples was done with the Kruskal-Wallis test. The maximum/minimum sCD26 concentration ratio was calculated for each patient to measure the sCD26 titer stability. ROC curves and areas under the curve (AUC) were calculated with this ratio using MedCalc (v.12.7.0). Data from sCD26, CEA, CA-19.9 and CA-72.4 for all the measurements during surveillance are presented on Table S2.

## Results

### Evolution of the cohort during the follow-up period

The average follow-up period for the 43 patients was 41.8±20.8 months, with a median of 34.1 months and a range of 9.7–79.6 months (Table S1). The 2 patients treated with palliative resection died during the study (mean 7.3±3.4 months). Regarding patients treated with curative resection, after the follow-up period 28 (patients 1–28) were disease-free (68.3%; mean follow-up: 44.9±19.5 months; range: 17.3–81.4 months), while local recurrences (patients 29–31) were documented in 3 cases (7.3%; mean follow-up: 25.7±3.1 months; range: 22.4–28.6 months). On the other hand, metastases were found in 10 patients (24.4%; mean follow-up: 44.7±22.2 months; range: 15.4–79.6 months), classified as 5 hepatic (patients 32–36), 3 pulmonary (patients 37–39), 1 peritoneal (patient 40) and 1 in jejunum and spleen (patient 41). All these metastases were diagnosed within 3 years after surgery, except for one liver metastasis diagnosed 4.3 years after surgery.

Chemotherapy was given to 29 patients: 17 free of disease, 2 with local recurrence and 10 with metastases.

### Tumor marker levels in preoperative blood samples

Preoperative serum samples were available for 41 patients (Table S1), 51.2% of which showed sCD26 levels below the 460 ng/mL cut-off point (21 cases). The clinical and epidemiological characteristics of these patients were analyzed according to two groups based on the normal levels described in the previous section: patients with positive (≤460 ng/mL or >850 ng/mL) and negative (460–850 ng/mL) preoperative sCD26. There were no significant differences in gender, age at diagnosis, Dukes’ stage, histological grade, tumor location, disease status or *exitus* between these groups (Table 1).

**Table 1:** Clinical and epidemiological characteristics of the patients according to the preoperative sCD26 levels.

Characteristic	Positive preoperative sCD26 (%)	Negative preoperative sCD26 (%)	*p* value
Male	18 (69.2%)	8 (30.8%)	0.102*
Female	6 (40.0%)	9 (60.0%)	
≤66 years	12 (54.5%)	10 (45.5%)	0.752*
>66 years	12 (63.2%)	7 (36.8%)	
Dukes A	4 (57.1%)	3 (42.9%)	0.999^+^
Dukes B	11 (57.9%)	8 (42.1%)	
Dukes C	6 (60.0%)	4 (40.0%)	
Dukes D	3 (60.0%)	2 (40.0%)	
Well differentiated	0 (0.0%)	1 (100.0%)	0.381^+^
Moderately differentiated	21 (63.6%)	12 (36.4%)	
Poorly differentiated	3 (50.0%)	3 (50.0%)	
Right colon	7 (53.8%)	6 (46.2%)	0.696^+^
Left colon	10 (55.6%)	8 (44.4%)	
Rectum	7 (70.0%)	3 (30.0%)	
Disease-free	17 (65.4%)	9 (34.6%)	0.249^+^
Local recurrence	1 (33.3%)	2 (66.7%)	
Distant metastasis	4 (40.0%)	6 (60.0%)	
Tumor persistence	2 (100.0%)	0 (0.0%)	
Local or distant recurrence	5 (38.5%)	8 (61.5%)	0.172*
No recurrence	17 (65.4%)	9 (34.6%)	
*Exitus*	8 (61.5%)	5 (38.5%)	1.000*
No *exitus*	16 (57.1%)	12 (42.9%)	

sCD26 levels were considered positive (≤460 or >850 ng/mL) or negative (460–850 ng/mL).

*p*-values correspond to: *Fischer’s exact test; ^+^Pearson’s Chi-square.

Regarding other preoperative markers, the CEA was determined in 33 patients, with 10 cases (30.3%) registering levels above the cut-off; CA-19.9 was analyzed in 21 cases, resulting over the cut-off in 3 cases (14.3%); and CA-72.4 appeared altered in 3 of 18 cases (16.7%). The clinical and epidemiological characteristics were also studied according to the positivity for each of these clinical markers (data not shown), and only the CA-72.4 and the histological grade of the tumor showed significant differences (*p* = 0.022).

### Tumor marker levels in postoperative blood samples

The levels of sCD26 and the clinical CRC markers were evaluated at the medical examinations attended by each patient during their surveillance. The maximum/minimum sCD26 concentration ratio is included in Table S1. Analysis of these measurements revealed trends, which allowed us to discriminate four groups of patients:

#### Disease-free patients at the end of the surveillance

**Figure 1:**
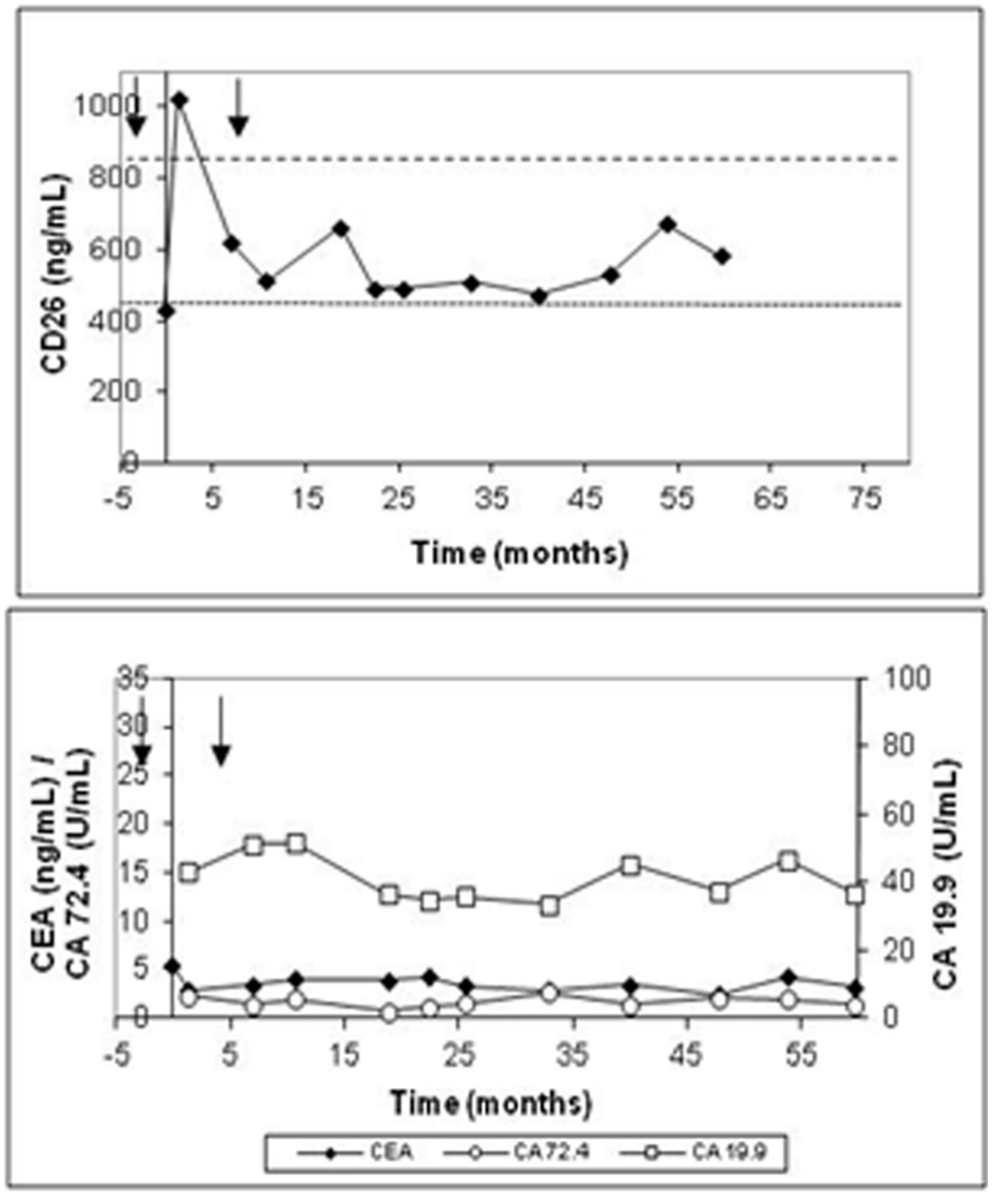
Levels of the sCD26, CEA, CA-19.9 and CA-72.4 in one representative disease-free patient (patient 25). Black arrows indicate beginning and end of chemotherapy cycles.

The general tendency followed by disease-free patients was the recovery of normal levels when preoperative sCD26 was low and stable titers above 460 ng/mL but below 850 ng/mL. Figure 1 shows the sCD26 levels during the follow-up of a representative patient (patient 25). This tendency was observed in 22 of the 28 disease-free patients (78.6%). Moreover, the group showed stable titers without important variations, resulting in a mean maximum/minimum sCD26 ratio of 1.52. Figure 1 also shows the values of CEA, CA-19.9 and CA-72.4 during the follow-up of the representative individual. All disease-free patients showed normal CEA levels in preoperative serum and throughout the surveillance time. CA-19.9 and CA-72.4 were not measured in 11 of these patients, but the data available showed that levels also tended to be stable and below the respective cut-off points, with only one individual (patient 25) showing increased CA-72.4 levels.

#### Patients with tumor persistence treated with palliative surgery

**Figure 2:**
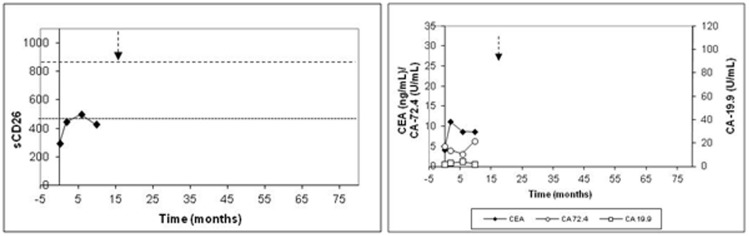
Levels of the sCD26, CEA, CA-19.9 and CA-72.4 in a representative individual (patient 42) with tumor persistence. The dashed arrow shows *exitus*.

The follow-up period for the two patients did not exceed 10 months due to their *exitus*. Low sCD26 levels were characteristic in this group, remaining fairly constant. In Figure 2 (patient 42), the sCD26 levels slightly rose over the 460 ng/mL threshold but decreased again to low values in the following measurement. The maximum/minimum sCD26 ratio resulted in 1.17 for this patient. Although the other patient also showed decreased sCD26 levels, only one measurement was made posterior to 2 months after surgery; therefore, the maximum/minimum ratio could not be calculated.

On the other hand, CEA, CA-19.9 and CA-72.4 measurements were available for one patient (Figure 2). During follow-up this individual showed increased CEA, while CA-19.9 and CA-72.4 displayed normal values.

#### Patients with recurrent tumors

**Figure 3:**
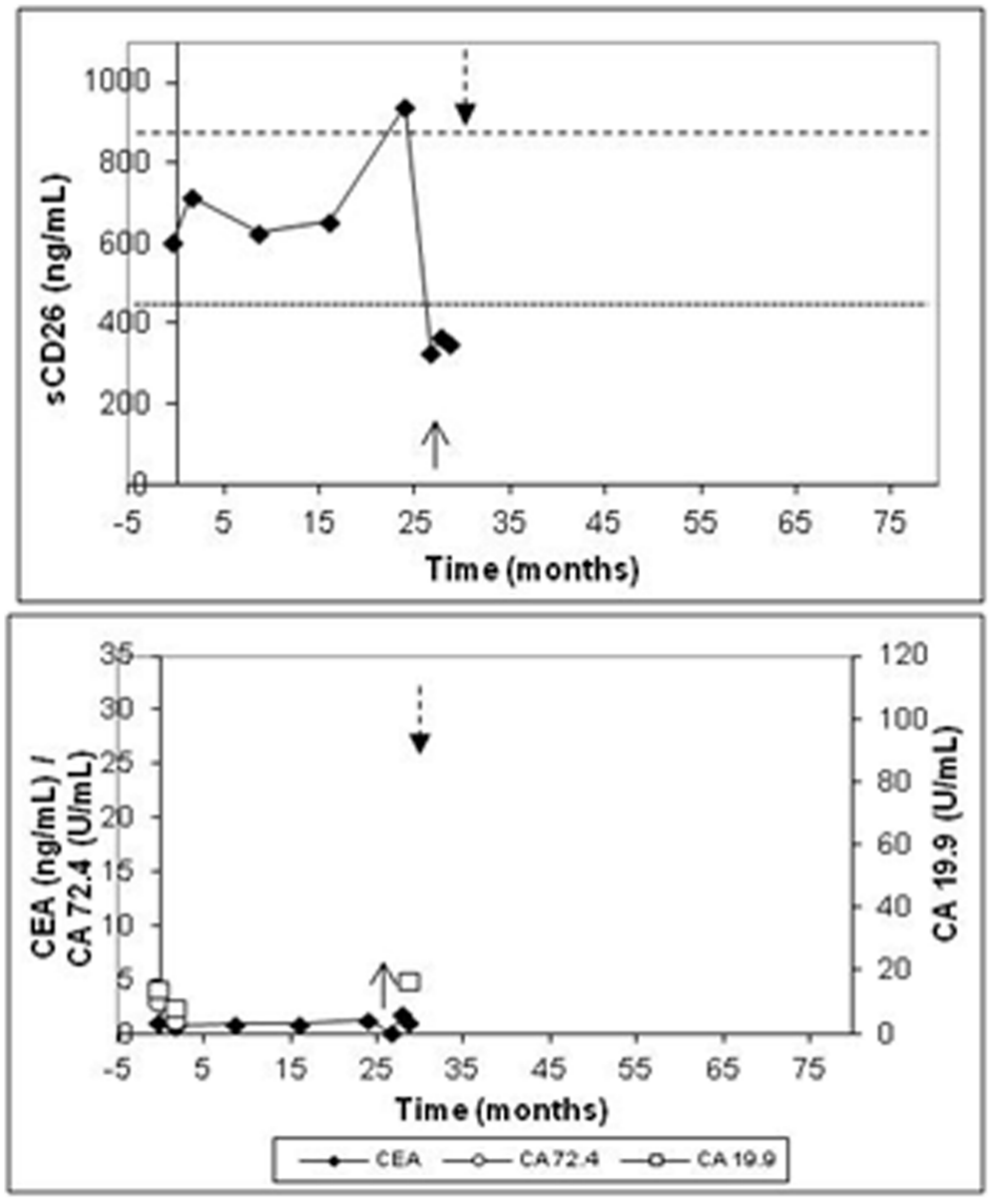
Levels of the sCD26, CEA, CA-19.9 and CA-72.4 in one representative patient with local recurrence (patient 31). The upwards arrow represents the diagnosis of recurrence, and the dashed arrow the time of *exitus*.

The 3 patients with recurrent tumors had a relapse time of 10.7, 25.4 and 26.6 months, respectively (patients 29–31). The evolution of the sCD26 levels is presented in Figure 3 for a representative individual (patient 31). In this case, during follow-up and before recurrence was diagnosed, the patients recovered normal levels (when low at the start). However, just before recurrence was confirmed, the three patients had a considerable increase (over 850 ng/mL in 2 of 3 cases), followed by one or two consecutive and acute decreases (not necessarily below 460 ng/mL). Therefore, instability in sCD26 levels preceded the appearance of recurrence. In relation to the maximum/minimum sCD26 ratio, it increased to 2.12.

Regarding the clinical markers, CEA was found elevated in 1 of the 3 cases, CA-19.9 also in 1 of the cases (Figure 3) and CA-72.4 in none of the patients.

#### Patients with metastatic disease

**Figure 4:**
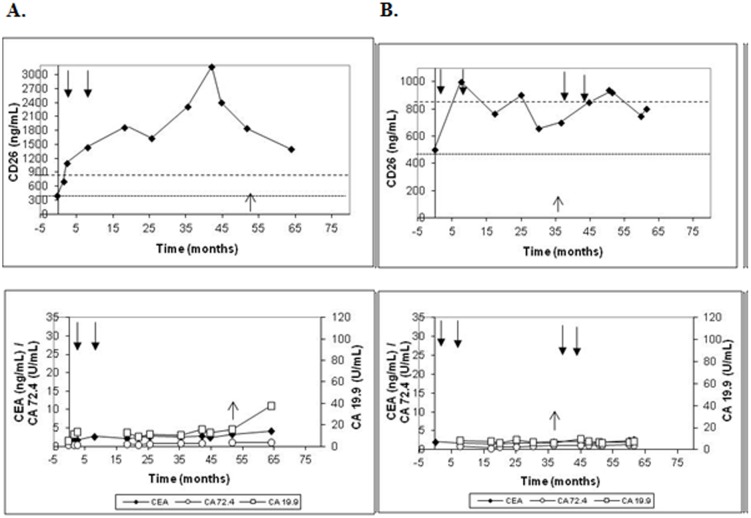
Levels of the sCD26, CEA, CA-19.9 and CA-72.4 in representative patients who developed hepatic (A; patient 34) and pulmonary (B; patient 39) metastases. Black arrows indicate beginning and end of chemotherapy cycles and the upwards arrow indicates diagnosis of metastasis.

The group of patients with metastasis showed another different trend. In the case of hepatic metastases (Figure 4A; patient 34), regardless of the preoperative sCD26 concentration, during follow-up levels reached or widely exceeded 850 ng/mL upper normal limit (in 4 of the 5 patients; 80.0%). In the other patient, we lacked samples from two years before the metastasis diagnosis but the last sample showed a value near that limit and the same trend to higher values. Therefore, the maximum/minimum sCD26 ratio for this sub-group corresponded to 2.17, similar to that for patients with local tumor recurrence.

In relation to the patients with pulmonary metastases (Figure 4B; patient 39) an important increase above the 850 ng/mL upper limit was detected before the metastases were diagnosed, followed by a decrease, suggesting unstable sCD26 levels. In this sub-group the maximum/minimum sCD26 ratio corresponded to 2.86, higher than the previous ratios. This trend was also found in patients with peritoneal or jejunum and spleen metastasis, with elevated sCD26 levels during follow-up, and a maximum/minimum sCD26 ratio of 1.48 (patient 40) and 5.34 (patient 41), respectively.

In summary, the general tendency observed in patients with metastasis was a sCD26 concentration over the upper 850 ng/mL cut-off, and an overall mean maximum/minimum sCD26 ratio of 2.63 for the group. To note, all of these patients had chemotherapy cycles, which seemed to low sCD26 levels transiently.

The behavior of the clinical markers in patients with metastasis is also shown in Figures 4A and 4B for representative patients. Despite the diagnosis of metastasis, CEA, CA-19.9 and CA-72.4 levels remained normal and stable during follow-up in 5/10, 4/10 and 7/10 patients, respectively.

### ROC curve analysis for postoperative serum sCD26

**Figure 5:**
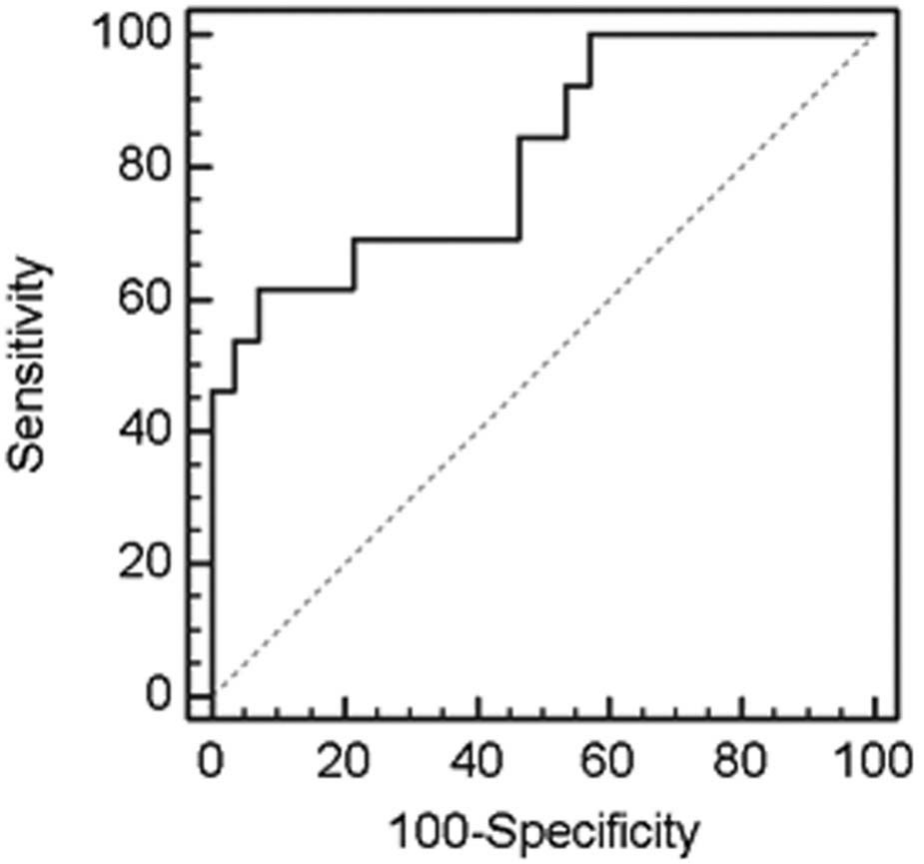
ROC curve for the maximum/minimum sCD26 ratio contrasting disease-free individuals and patients with local or distant recurrence. The corresponding AUC is provided in the text.

Statistically significant differences were observed in the maximum/minimum sCD26 ratio between disease-free patients, patients with local recurrence and those with metastasis (*p* = 0.005). These differences were further studied using ROC curve analysis (generated with the maximum/minimum sCD26 ratios). Only patients treated by curative surgery were included, classified as disease-free (n = 28) or with local or distant recurrence (n = 13). An AUC of 0.835 (95% CI 0.702–0.968; *p*<0.0001) (Figure 5) was obtained, showing an optimal accuracy for separating patients with recurrent disease. 100% sensitivity was obtained with a maximum/minimum sCD26 ratio cut-off of 1.43 (46.4% specificity). Specificity can be further enhanced taking into account that most false positive disease-free patients did not overcome the 850 ng/mL cut-off. However, a higher specificity (92.9%) was observed for the 1.98 cut-off (61.5% sensitivity).

## Discussion

Numerous studies, systematic reviews and meta-analyses, as well as the American and European cancer societies, conclude that the best way to accomplish the early diagnosis of recurrence and improve survival is through intensive surveillance. However, consensus has not been achieved regarding the protocol for intensive follow-up (combination of tests and frequency) [1]–[8].

In fact, the currently used clinical markers [9], [10], including CA-19.9 and CA-72.4, are neither recommended for surveillance following curative resection nor for prognosis [5]–[8]. In their guidelines, both the EGMT and ASCO groups recommend the measurement of CEA in CRC patients in stage II or III every 2–3 months [6] or every 3 months [8] during at least 3 years after diagnosis. However, there is a continuous debate around CEA mainly due to its lack of specificity. Some authors concluded that a rise in the antigen concentration is a poor predictor of local recurrence, and even in patients with liver metastases a rising concentration is a relative late phenomenon [21], [22]; consequently, serum CEA should be abandoned in routine follow-up [6]. Our results in this study agree with this recommendation.

In our previous studies, low serum sCD26 levels were observed in CRC patients from different cohorts: a case-control cohort [15] and a mainly symptomatic cohort who underwent colonoscopy [16]. In the first study, different levels of preoperative sCD26 could be associated to increased risk of developing a recurrent disease [15]. Additionally, we as well as others observed that some metastatic CRC patients showed high sCD26 concentrations [16], [20]. In this pilot study we measured both the preoperative and the postoperative sCD26 levels to assess its capability in predicting and anticipating the diagnosis of a recurrent disease either locally or in a distant organ. As 80% of all recurrences are diagnosed within the first two years after surgery [8], [22], we undertook a follow-up during at least this period.

The clinical and epidemiological characteristics of the patients (gender, age, Dukes’ stage, differentiation or localization of the tumor) analyzed according to the preoperative sCD26 levels rendered no association, corroborating our previous findings [15]. Although stage D patients showed higher levels compared to less invasive tumor stages, as previously observed [15], no statistically significant differences were found for the disease status or the presence/absence of recurrence in relation to sCD26 positivity based on the 460/850 ng/mL cut-off.

In relation to the sCD26 performance considering preoperative samples, we found a reduced sensitivity for the diagnosis of CRC in this cohort (51.2%), compared to the 81.8% observed previously [16] or by others [17], [18]. Differences may be attributed to study settings since in this cohort only patients already diagnosed of CRC were included, while in the other study mainly symptomatic individuals diagnosed of diverse colorectal pathologies were included. Alternatively, technical reasons related to the specificity of antibodies used in the Elisa for sCD26 detection may also explain the differences [14]. In relation to this, and as discussed elsewhere [20], the sCD26 cut-off was changed from 410 in our first study [15] to the 460 ng/mL [16] used here. Thus, based on our previous results with this kit [15], [16], [19] sCD26 values between 460–850 ng/mL were considered normal. We preliminarily chose the 850 ng/mL upper limit for this study because in our previous works sCD26 levels were found within this range in non-pathological individuals (reviewed in [20]). Also, the amount of DPP-IV/CD26 antigen found in normal serum is consistent with the expected values based on the specific activity of purified serum DPP-IV [23] and there is usually correlation between DPP-IV activity and sCD26 levels in pathological conditions [21].

Based on the proposed sCD26 normal range in the postoperative measurements, we were able to define different characteristic trends for the disease status. These were further confirmed with a ROC curve based on the maximum/minimum sCD26 ratio that measured sCD26 titer stability during surveillance. This analysis showed an optimal accuracy for distinguishing disease-free patients from those with local or distant recurrent disease. According to the above, in most disease-free patients stable sCD26 levels (460–850 ng/mL; maximum/minimum ratio 1.52) were found. Increases over 850 ng/mL were detected in 6 disease-free patients (2 cases registered increases at the end of the surveillance period and no further information about changes in disease status could be obtained; 2 cases were diagnosed of space-occupying lesions in the liver with no evidence of hepatic metastasis; while no explanation for high levels were found in the other 2 cases).

In relation to the patients with local or distant recurrence, in the majority of the cases (10/13; 76.92%) sCD26 levels surpassed the 850 ng/mL and were unstable (maximum/minimum ratio 2.49). Specifically, sudden increases above 850 ng/mL followed by consecutive and acute decreases could predict recurrence at least 2–3 months before the clinical diagnosis in the 3 patients with local recurrence. This would translate into an earlier oncological treatment and surgical resection, with an increased survival rate [3]–[5].

In the case of distant recurrences, liver and lung were the most frequent organs affected in our cohort, consistent with 35–55% of hepatic metastasis and 10% of lung metastasis reported for CRC patients [24]–[25]. Hepatic metastases at the initial diagnosis were detected in 3 patients and during follow-up in 2 patients. In one of the latter, high sCD26 concentrations (reaching 3.200 ng/mL) were observed from 2 months post-surgery and during all the follow-up, indicating at a very early stage (more than 49 months ahead) the suspicion of metastasis. On the contrary, in the other patient, levels below the 460 ng/mL cut-off were found 11 months before confirmation of metastasis, also indicating the presence of recurrence. In two of the patients with lung metastasis, increases in sCD26 over 850 ng/mL were registered 3.8 and 29.1 months, respectively, before the diagnosis of metastasis; in the other case, elevated levels (2.900 ng/mL) coincided with the diagnosis. Once again, our test would have anticipated the diagnosis of metastasis.

Our findings suggest that the periodic measurement of serum sCD26 levels every 3 months could serve as guide for oncological decision-making, alerting about the appearance of recurrence based on the maximum/minimum sCD26 ratio and the sCD26 levels during surveillance. The behavior of sCD26 according to the disease status is summarized on Figure S1. Nonetheless, these results should be regarded as preliminary and should be extended to a larger dataset in further prospective and retrospective studies. Yet, the implementation of this test to the clinical practice could be feasible since a blood extraction is done regularly during follow-up of CRC patients and the test consists of a typical Elisa assay.

With respect to the biological significance of our results, we have reviewed that, pathophysiological, low sCD26 levels occur concurrently with an impaired immune status, including some hematological and solid malignancies, whereas increased levels occur in inflammatory and infectious diseases, other hematological tumors, and liver diseases such as hepatocellular carcinoma [14]. The soluble sCD26 found in serum is presumably shed by proteolytic cleavage of the transmembrane CD26 [14], [26]. Besides the classical capillary endothelial, hepatic and immune tissues from which sCD26 could originate [13], [14], [26], recently the adipose tissue [27] and muscle [28] may also be included.

It is now clear that immune-related mechanisms are skills that cancer cells should acquire on their way to giving rise to a tumor, including the ability to thrive in a chronically inflamed microenvironment, the ability to evade immune recognition and the ability to suppress immune reactivity. These three capabilities have been recognized recently as the immune hallmarks of cancer [29]. Hence, for CRC we have hypothesized [14] the immune system as the source of the impaired levels mainly because CD26 is not differentially expressed in primary tumors and normal colon tissues [30], [31]. In addition, many *in vivo* studies found a correlation between changes in serum DPP-IV activity and the numbers of PBL, T lymphocytes, CD26+ T cells and the amount of CD26 in T lymphocyte plasma membranes (reviewed in [14]). Therefore, it may be possible that the developing tumor may be immunosuppressing a sCD26-generating population or down regulating the production of circulating sCD26 through TGF-β [32].

Interestingly, the elevated sCD26 concentrations found in Dukes D CRC patients with metastasis [15] and in this work may be related with the recent findings from Pang and colleagues [31]. They reported differential expression of CD26 between primary tumors and metastases. These authors identified the CD26-expressing cells as cancer stem cells (CSC), associated with enhanced invasiveness and chemoresistance. When isolated and injected into mice these CD26+ cells led to the development of distant metastasis [31]. If these cells are producing increased levels of sCD26 it may be related to the quick expansion of the population or perhaps because of an increased metabolism of CD26 expression and shedding. This idea agrees and complements our findings of elevated sCD26 levels in metastatic patients. In line with the previous report, a recently published work from Lam and colleagues [33] reported significantly higher tumor CD26 expression levels in CRC patients with distant metastasis compared to non-metastatic patients. Additionally, *in vitro* experiments with these CSC are ongoing in our lab to analyze their ability to produce sCD26, which could probably explain the elevated sCD26 concentrations found in metastatic patients.

No doubt these changes have important consequences in oncogenic processes. Current data supports three potential roles of sCD26 in: (i) activation–deactivation of chemokines in inflammatory processes; (ii) activation-inactivation of other biologically active blood substrates, growth factors or hormones; and (iii) cell-adhesion, migration and invasion capacities [12]–[14], [31], [34], [35].

## Conclusion

Serum sCD26 levels showed well-defined patterns during follow-up of CRC patients. Stable sCD26 concentrations were characteristic in disease-free patients; while patients with local or distant recurrent disease showed elevated sCD26 levels with sudden decreases, resulting in instability. The measurement of sCD26 may help to accomplish an early detection of recurrent CRC disease after surgery, even in patients under chemotherapy. Once confirmed in a larger prospective trial, sCD26 could be a valuable marker for postoperative surveillance.

## Supporting Information

Figure S1 **Schematic representation of the behavior of sCD26 during follow-up of CRC patients according to the disease status.**
(TIF)

Table S1 **Clinical characteristics of the cohort followed-up in the study.**
(DOCX)

Table S2 **Serum levels of sCD26, CEA, CA-19.9 and CA-72.4 for preoperative and postoperative** **measurements.**
(XLS)
