# Supplementary figures and images for: Postoperative Serum Levels of sCD26 for Surveillance in Colorectal Cancer Patients
Source: PLoS One. 2014 Sep 11;9(9):e107470. doi: 10.1371/journal.pone.0107470 (PMC4161426; doi:10.1371/journal.pone.0107470)

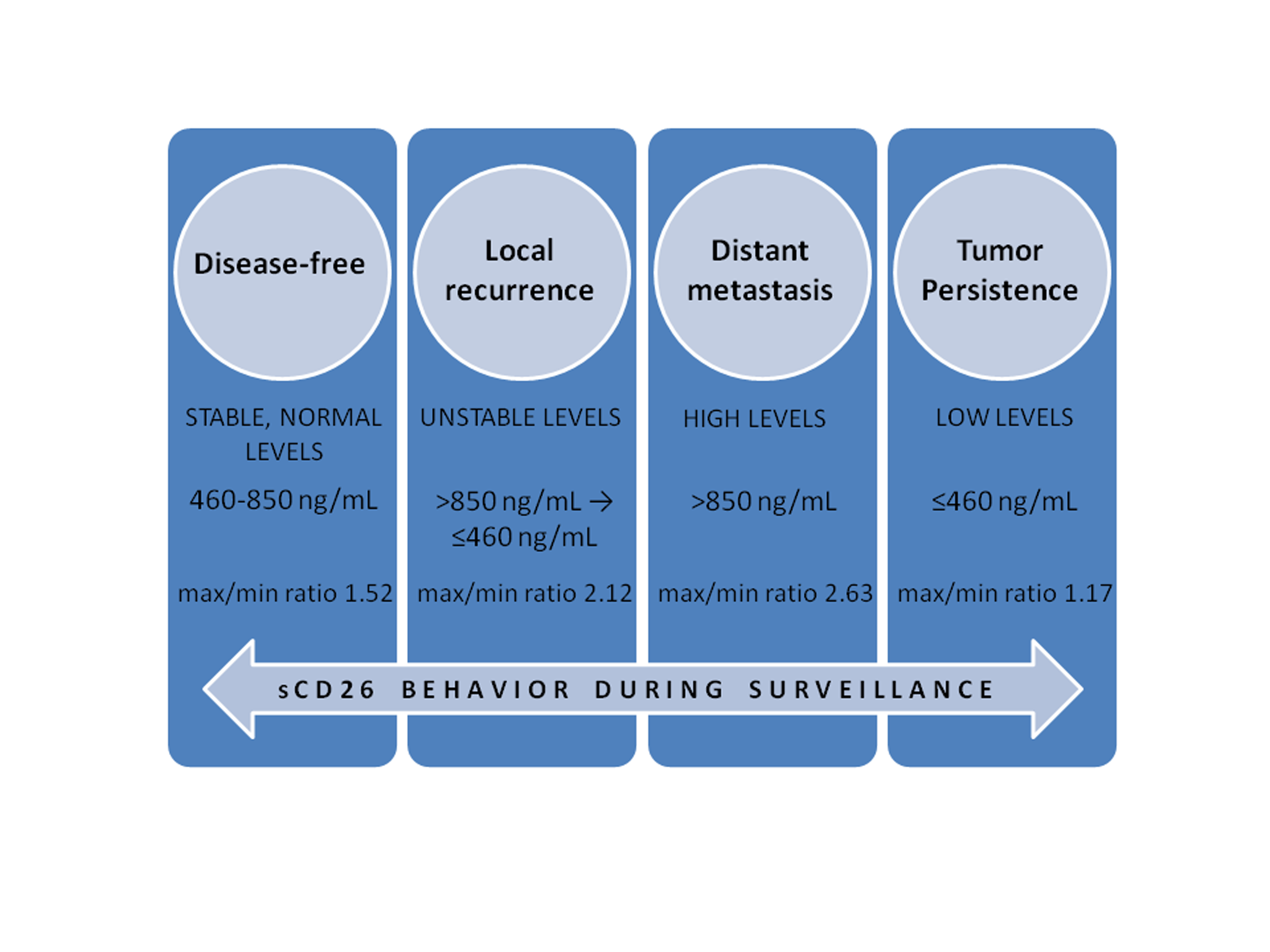

Supplement: Figure S1 — Schematic representation of the behavior of sCD26 during follow-up of CRC patients according to the disease status. (TIF) [file pone.0107470.s001.tif]
