# Supplementary material for: Postoperative Serum Levels of sCD26 for Surveillance in Colorectal Cancer Patients
Source: PLoS One. 2014 Sep 11;9(9):e107470. doi: 10.1371/journal.pone.0107470 (PMC4161426; doi:10.1371/journal.pone.0107470)
Supplement: Table S1 — Clinical characteristics of the cohort followed-up in the study. (DOCX) [file pone.0107470.s002.docx]

**Table S1.** Clinical characteristics of the cohort followed-up in the study.

| **Patient** | **Dukes’ stage** | **Primary tumor site** | **Resection** | **Chemotherapy** | **Follow-up (months)^1^** | ***Exitus* (month)^1^** | **Pre-sCD26^2^** | **Min./Max. sCD26 ratio^3^** | **Disease status^4^** |
| --- | --- | --- | --- | --- | --- | --- | --- | --- | --- |
| 1 | B | Hepatic flexure | Curative | Yes | 39.7 |  | - | 1.32 | DF |
| 2 | A | Rectum | Curative | No | 29.6 | 95.0 | 451.26 | 1.66 | DF |
| 3 | B | Sigma | Curative | Yes | 36.0 |  | 501.49 | 1.34 | DF |
| 4 | C | Cecum, transverse colon | Curative | Yes | 33.6 |  | 325.08 | 1.60 | DF |
| 5 | C | Sigma | Curative | Yes | 29.9 |  | 299.15 | 1.48 | DF |
| 6 | A | Rectum | Curative | No | 35.3 |  | 510.32 | 1.18 | DF |
| 7 | B | Hepatic flexure | Curative | Yes | 32.4 |  | 555.47 | 1.69 | DF |
| 8 | B | Rectum | Curative | Yes | 32.0 |  | 459.23 | 1.54 | DF |
| 9 | B | Sigma | Curative | Yes | 25.1 |  | 491.38 | 1.20 | DF |
| 10 | C | Rectum-sigma | Curative | Yes | 26.3 | 68.7 | 124.49 | 1.62 | DF |
| 11 | B | Sigma | Curative | Yes | 25.1 |  | 417.82 | 1.30 | DF |
| 12 | C | Splenic flexure | Curative | Yes | 19.2 |  | - | 1.25 | DF |
| 13 | A | Rectum-sigma | Curative | No | 22.8 |  | 435.52 | 1.23 | DF |
| 14 | B | Descending colon | Curative | Yes | 17.3 |  | 508.73 | 1.13 | DF |
| 15 | B | Rectum-sigma | Curative | Yes | 31.0 | 35.0 | 347.33 | 2.09 | DF |
| 16 | B | Rectum | Curative | No | 77.9 |  | 393.33 | 1.49 | DF |
| 17 | C | Cecum | Curative | Yes | 81.4 |  | 400.67 | 1.41 | DF |
| 18 | B | Rectum | Curative | Yes | 73.5 |  | 1,146.67 | 2.25 | DF |
| 19 | B | Rectum-sigma | Curative | No | 63.2 |  | 665.33 | 1.63 | DF |
| 20 | A | Rectum | Curative | No | 63.6 |  | 448.74 | 1.29 | DF |
| 21 | B | Rectum-sigma | Curative | No | 66.6 |  | 516.67 | 1.66 | DF |
| 22 | B | Hepatic flexure | Curative | Yes | 61.4 |  | 191.20 | 1.72 | DF |
| 23 | B | Descending colon | Curative | No | 59.0 |  | 855.20 | 1.71 | DF |
| 24 | A | Cecum | Curative | No | 64.4 |  | 497.42 | 1.76 | DF |
| 25 | C | Rectum | Curative | Yes | 59.6 |  | 432.35 | 1.42 | DF |
| 26 | B | Ascending colon | Curative | No | 60.3 |  | 785.60 | 1.26 | DF |
| 27 | B | Cecum | Curative | Yes | 34.1 | 49.0 | 386.88 | 1.93 | DF |
| 28 | B | Sigma | Curative | No | 56.6 |  | 411.62 | 1.49 | DF |
| 29 | B | Hepatic flexure | Curative | Yes | 26.1 |  | 268.00 | 1.78 | LR |
| 30 | C | Hepatic flexure | Curative | Yes | 22.4 |  | 623.00 | 1.69 | LR |
| 31 | A | Rectum | Curative | No | 28.6 | 29.4 | 604.00 | 2.88 | LR |
| 32 | D | Sigma | Curative | Yes | 15.4 | 21.3 | 740.08 | 1.44 | DM |
| 33 | D | Hepatic flexure | Curative | Yes | 79.6 |  | 696.00 | 2.22 | DM |
| 34 | B | Hepatic flexure | Curative | Yes | 63.9 |  | 406.67 | 2.90 | DM |
| 35 | D | Sigma | Curative | Yes | 35.1 | 37.4 | 1,060.40 | 2.04 | DM |
| 36 | C | Sigma | Curative | Yes | 51.9 |  | 453.00 | 2.26 | DM |
| 37 | A | Rectum | Curative | Yes | 31.8 | 31.0 | 380.67 | 2.85 | DM |
| 38 | B | Rectum | Curative | Yes | 62.0 | 66.2 | 587.43 | 4.21 | DM |
| 39 | C | Sigma | Curative | Yes | 61.3 |  | 502.65 | 1.53 | DM |
| 40 | C | Ascending colon, cecum | Curative | Yes | 14.4 | 17.8 | 787.43 | 1.48 | DM |
| 41 | C | Splenic flexure | Curative | Yes | 31.6 | 34.0 | 550.67 | 5.34 | DM |
| 42 | D | Cecum | Palliative | No | 9.7 | 17.8 | 290.90 | 1.17 | TP |
| 43 | D | Sigma | Palliative | No | 4.9 | 5.4 | 277.30 | - | TP |

^1^The date of surgery is considered as time 0.

^2^Pre-sCD26 refers to the preoperative measurement of the marker.

^3^Min/Max sCD26 ratio refers to the maximum/minimum sCD26 concentration ratio calculated for each patient.

^4^For each patient the disease status during follow-up is indicated: disease-free (DF; patients 1-28), local recurrence (LR; patients 29-31), distant metastasis (DM; patients 32-41) or tumor persistence (TP; patients 42-43).
